# *Pseudomonas aeruginosa* Population Structure Revisited

**DOI:** 10.1371/journal.pone.0007740

**Published:** 2009-11-13

**Authors:** Jean-Paul Pirnay, Florence Bilocq, Bruno Pot, Pierre Cornelis, Martin Zizi, Johan Van Eldere, Pieter Deschaght, Mario Vaneechoutte, Serge Jennes, Tyrone Pitt, Daniel De Vos

**Affiliations:** 1 Laboratory for Molecular and Cellular Technology, Burn Centre, Queen Astrid Military Hospital, Brussel, Belgium; 2 Applied Maths, Sint-Martens-Latem, Belgium; 3 Laboratory of Microbial Interactions, Department of Molecular and Cellular Interactions, Flanders Interuniversity Institute of Biotechnology, Vrije Universiteit Brussel, Brussel, Belgium; 4 Department of Physiology, Vrije Universiteit Brussel, Brussel, Belgium; 5 Rega Institute and Laboratory of Microbiology, Gasthuisberg University Hospital, Catholic University of Leuven, Leuven, Belgium; 6 Laboratory for Bacteriology Research, Department of Chemistry, Microbiology and Immunology, Ghent University, Ghent, Belgium; 7 Laboratory of Healthcare Associated Infection, Specialist and Reference Microbiology Division, Health Protection Agency, London, United Kingdom; University of Hyderabad, India

## Abstract

At present there are strong indications that *Pseudomonas aeruginosa* exhibits an epidemic population structure; clinical isolates are indistinguishable from environmental isolates, and they do not exhibit a specific (disease) habitat selection. However, some important issues, such as the worldwide emergence of highly transmissible *P. aeruginosa* clones among cystic fibrosis (CF) patients and the spread and persistence of multidrug resistant (MDR) strains in hospital wards with high antibiotic pressure, remain contentious. To further investigate the population structure of *P. aeruginosa*, eight parameters were analyzed and combined for 328 unrelated isolates, collected over the last 125 years from 69 localities in 30 countries on five continents, from diverse clinical (human and animal) and environmental habitats. The analysed parameters were: i) O serotype, ii) Fluorescent Amplified-Fragment Length Polymorphism (FALFP) pattern, nucleotide sequences of outer membrane protein genes, iii) *oprI*, iv) *oprL*, v) *oprD*, vi) pyoverdine receptor gene profile (*fpvA* type and *fpvB* prevalence), and prevalence of vii) exoenzyme genes *exoS* and *exoU* and viii) group I pilin glycosyltransferase gene *tfpO*. These traits were combined and analysed using biological data analysis software and visualized in the form of a minimum spanning tree (MST). We revealed a network of relationships between all analyzed parameters and non-congruence between experiments. At the same time we observed several conserved clones, characterized by an almost identical data set. These observations confirm the nonclonal epidemic population structure of *P. aeruginosa*, a superficially clonal structure with frequent recombinations, in which occasionally highly successful epidemic clones arise. One of these clones is the renown and widespread MDR serotype O12 clone. On the other hand, we found no evidence for a widespread CF transmissible clone. All but one of the 43 analysed CF strains belonged to a ubiquitous *P. aeruginosa* “core lineage” and typically exhibited the *exoS* ^+^/*exoU* ^−^ genotype and group B *oprL* and *oprD* alleles. This is to our knowledge the first report of an MST analysis conducted on a polyphasic data set.

## Introduction

In his 1882 paper, “Sur les colorations bleue et verte des linges à pansements”, introduced by Louis Pasteur, Carle Gessard describes the isolation of an organism causing a blue-green coloration of wound dressings [1]. He describes this ‘accidental’ organism as colourless, globular, 1 to 1.5 thousandths of a millimetre in length, aerobic and very motile. The bacterium was named *Bacillus* (rod) *pyocyaneus*. Today we refer to this organism as *Pseudomonas aeruginosa*. This species is ubiquitous in the biosphere, has wide metabolic versatility and high intrinsic and acquired resistance to antimicrobials. It can be found in a wide variety of ecological environments ranging from fresh and salt water to the rhizosphere in which they colonize the endemic fauna (e.g. nematodes), flora and fungi (e.g. *Pythium* spp.) [2]. The opportunistic bacterium *P. aeruginosa* occasionally migrates from its natural environment and causes disease in animals (wild, domestic and livestock) and humans. In the latter it has emerged, partly due to its intrinsic antibiotic resistance, as a major pathogen in the airways of cystic fibrosis (CF) patients, causing often-fatal chronic respiratory infections, and as one of the most clinically significant opportunist nosocomial agents. Immunosuppressed patients such as those with severe burns, cancer or AIDS are particularly at risk.

Numerous research groups have demonstrated that *P. aeruginosa* clinical isolates are genotypically, chemotaxonomically, and functionally indistinguishable from environmental isolates. Römling *et al*. observed that the most frequently identified clone in CF patients was also detected at a relatively high frequency in aquatic environments [3] and Rahme *et al*. demonstrated the infectivity of a *P. aeruginosa* strain in both plant and animal models [4]. Similarly, *P. aeruginosa* strains isolated from a gasoline-contaminated aquifer were indistinguishable from clinical isolates [5] and both oil-contaminated soil isolates and clinical isolates showed pathogenic and biodegradative properties [6].

### Population structure

Using multilocus enzyme electrophoresis, Maynard Smith and colleagues demonstrated that bacterial population structures could range from panmictic or fully sexual, with random association between alleles, to clonal, with nonrandom association of alleles, the latter resulting in the frequent recovery of relatively few of the many possible multilocus genotypes [7]. An intermediate type of population structure that is predominantly sexual, but harbours some epidemic clones, which show significant association between loci, was called ‘epidemic’.

The population structure of *P. aeruginosa* has been the subject of numerous investigations, we present an overview. Both Denamur *et al*. in 1993, and Picard *et al*. in 1994, suggested a panmictic population structure for the species but highlighted the need for caution in inferring the population structure from any single class of genetic marker [8], [9]. In 2000, comparative sequencing of 19 environmental and clinical isolates revealed a net-like population with a high frequency of recombination between isolates [10]. Using randomly amplified polymorphic DNA typing, Ruimy *et al*. demonstrated that bacteremia and pneumonia were not caused by specific *P. aeruginosa* clones [11]. In 2001 Lomholt and colleagues suggested an epidemic population structure for a *P. aeruginosa* population isolated mainly from patients with keratitis and their environment [12]. They found evidence for an epidemic clone that is pathogenic to the eye and is characterized by a distinct combination of virulence factors. In 2002, we combined the data obtained by 4 different typing methods, performed on a batch of 73 unrelated clinical and environmental *P. aeruginosa* isolates, collected across the world and observed a clear mosaicism in the results and a non-congruence between experiments, features of a panmictic population structure [13]. But, in this network we also observed some clonal complexes characterized by an almost identical data set. There was no obvious correlation between these dominant clones and habitat or, with the exception of some recent clones, their geographical origin. Therefore, we suggested an epidemic population structure for *P. aeruginosa*. Using multi locus sequence typing (MLST), Curran *et al*. confirmed in 2004 that *P. aeruginosa* exhibits a nonclonal epidemic population structure [14]. The *P. aeruginosa* population in the River Woluwe in Brussels was found to be almost as diverse as the global population, harbouring members of nearly all successful clonal complexes [15].

Several groups found that *P. aeruginosa* possessed a highly conserved genome, which encoded genes important for survival in numerous environments including humans and evolved through the acquisition, loss, and reorganisation of genome islands and genome islets [16]–[20]. Horizontal gene transfer (HGT) might play a more important role than point mutation in the adaptation of *P. aeruginosa* to different habitats. Despite not believed to be naturally competent, *P. aeruginosa* displays a high level of interstrain genomic plasticity and contains a high number of unfixed genes. Shen *et al*. put forward the idea of a population-based supra-genome that is substantially larger than the genome size of any of the component strains [21]. No two strains would be identical in terms of their genetic content and HGT continuously creates new strains with unique genetic characteristics.

Environmentally endemic bacteriophages are probably responsible for a fair amount of HGT, as they were shown to be formidable transducers of naturally occurring microbial communities of *P. aeruginosa* [22].

In 2006 Lee and colleagues tested the pathogenicity of diverse *P. aeruginosa* strains in a *Caenorhabditis elegans* pathogenicity model and showed that genes required for pathogenicity in one strain of *P. aeruginosa* were neither required for, nor predictive of virulence in other strains [23]. They concluded that virulence in *P. aeruginosa* is both multifactorial and combinatorial, the result of a pool of pathogenicity-related genes that interact in various combinations in different backgrounds.

In 2007 Wiehlmann and colleagues analysed 240 *P. aeruginosa* strains with a DNA array tube assay and reported the segregation of strains from diverse habitats and geographic origin into two large nonoverlapping clusters and 45 isolated clonal complexes composed of a few or even single strains [24]. The majority of strains belonged to a few dominant clones widespread in disease and environmental habitats.

In conclusion, there appears to be a consensus that the *P. aeruginosa* population structure is nonclonal epidemic, that clinical isolates are indistinguishable from environmental isolates, and that there are no specific clones with a specific (disease) habitat selection. The *P. aeruginosa* genome consists of a highly conserved core spiked with mobile islands and elements, which are exchanged between strains through intensive and basically phage-mediated HGT, thus creating the striking diversity of this ubiquitous opportunistic pathogen.

Despite the above-mentioned studies, some important contentious issues remain. First, since the 1980s several studies have reported the emergence, spread and persistence of multidrug resistant (MDR) clones in hospitals, mainly in intensive care wards with high antibiotic pressure. Two serotypes, O11 and O12, are highly associated with these epidemic strains [25]–[47]. Typing of these strains supported a heterogeneous population in serotype O11 but those of serotype O12 often appeared to lack significant diversity.

Second, since the second half of the 1990s, an increasing number of *P. aeruginosa* ‘transmissible’ CF clones have been reported worldwide [48]–[60], suggestive of an emergence of specific clones that have adapted to the CF airway environment and are spreading within CF populations.

### This study

To provide a reference evolutionary framework and to position these emergent *P. aeruginosa* clones in the global population structure, we decided to expand our earlier study [13] both in terms of number and range of isolates and of characters investigated. Our starting material consisted of a collection of 328 unrelated isolates, collected over the last 125 years from 69 localities in 30 countries on 5 continents, including isolates from diverse clinical (human and animal) and environmental habitats (Table 1).

**Table 1:** Origin of the *P. aeruginosa* strains (summary).

Locality	Country	CF	Clinical non CF	Animal	Environment	Hospital environment	Unknown
Buenos Aires	Argentina		1				
Hobart	Australia	2	15		2	1	
Melbourne	Australia	1	1				
Antwerp	Belgium	2					
Brussels	Belgium	9	13		13	6	
De Haan Holiday Camp	Belgium	4					
Geel	Belgium		1				
Ghent	Belgium	5	4		1		
Leuven	Belgium	3	2				
North sea	Belgium				1		
Cotonu	Benin				1		
Sofia	Bulgaria		5			1	
Vancouver	Canada	2					
Chengdu	China						1
Cali	Colombia		3			1	
Prague	Czech Republic		1			2	1
Lwiro	Democratic Republic of Congo		2				
Nantes	France		1				
Paris	France		14	1			
Tbilisi	Georgian Republic		2				
Hanover	Germany	7				2	
Mulheim	Germany				2		
Ruhr River	Germany				1		
Aachen	Germany		3				
Athens	Greece		5				
Budapest	Hungary		3				2
IDEXX Laboratories	India			11			
Pordenone	Italy		1				
Lake Tamako	Japan				2		
Otshuchi Bay	Japan			1			
Pacific Ocean	Japan				10		
Sagami Bay	Japan				3		
Zenpukujii Pond	Japan				1		
Arakawa River	Japan				2		
Suruga Bay	Japan				2		
Loltun	Mexico				1		
Karachi	Pakistan				2		
Panama City	Panama		10				
Lisbon	Portugal	4	1				
Veterinary	Portugal			35			
Canas	Puerto Rico				1		
Unknown	Puerto Rico				1		
Bucarest	Roumania		7		1		1
Beverwijk	The Netherlands		4				
Holiday Camps	The Netherlands	1					
Rotterdam	The Netherlands		1				
Tacloban City	The Philippines		1				
Unknown	The Philippines				1		
Mediterranean sea	Tunisia				1		
Tunis	Tunisia		3				
Istanbul	Turkey		6				
Birmingham	UK	1					
Cambridge	UK				1		
Colindale	UK		1				
Elstree	UK		3				
IDEXX Laboratories	UK			10			
Liverpool	UK	1					
London	UK		9				
Manchester	UK	1					
NIMR	UK						2
Roehampton	UK		1				
Surrey	UK		1				
Ann Arbor	US		3				
Boston	US		5				
California	US						2
Detroit	US						1
Jekyll Island	US			5	5		
Kentucky	US						2
San Antonio	US		9				
69 localities	30 countries (5 continents)	**43**	**142**	**63**	**55**	**13**	**12**

Since different (genetic) markers have been shown to measure different evolutionary forces, confirming the importance of a polyphasic approach to population analysis [8], [9], [19], [61], [62], we decided to analyse and combine data from eight parameters that are equally dispersed over the *P. aeruginosa* genome (Table 2). The parameters investigated were i) O-serotype, ii) total genome profile by fluorescent amplified-fragment length polymorphism (FAFLP) analysis, nucleotide sequence of the outer membrane protein genes iii) *oprI*, iv) *oprL*, and v) *oprD*, vi) pyoverdine receptor gene profile (*fpvA* type and *fpvB* prevalence), and the prevalence of vii) exoenzyme genes *exoS* and *exoU* and viii) group I pilin glycosyltransferase gene *tfpO*.

**Table 2:** Genomic localisation of the parameters investigated in this study.

Gene	Genomic localization (Mb)*
*oprD*	∼1.04
*oprL*	∼1.06
*fpvA*	∼2.66
*oprI*	∼3.21
Serotype (*wbpM-himD*)	∼3.53
*exoS*	∼4.30
*exoU*	∼4.58
*fpvB*	∼4.66
*tfpO* (*pilO*)	∼5.07

*Localisation in the genome of reference strain PAO1 (reference strain UCBPP-PA14 for *exoU*) according to the *Pseudomonas aeruginosa* Genome Database (http://www.pseudomonas.com).

Serotyping only allows for a crude discrimination between different *P. aeruginosa* isolates, but because it has been performed all over the world for more than 80 years [63] it forms a bridge between old and new epidemiological studies.

FAFLP is a highly discriminatory and reproducible genotyping method based on the selective amplification of a subset of DNA fragments generated by restriction enzyme digestion [64]–[66].

Although it is generally assumed that the best means of indexing natural variation in a population structure is to sequence housekeeping genes [67] we previously showed that the DNA sequence of the *oprI*, *oprL* and *oprD* genes generated equally discriminative data [13]. The *oprI* and *oprL* genes, which code for outer membrane lipoproteins [68]–[71], showed sequence diversity comparable to that of housekeeping genes [13] and have been included in SNP schemes [19].

The *P. aeruginosa oprD* gene codes for a specialized pore protein, OprD, which allows selective permeation of basic amino acids and their structural analogs like the carbapenem antibiotics imipenem and meropenem [72], [73]. It exhibits important sequence variability with multiple non-silent mutations and a microscale mosaic structure resulting from multiple recombinational events [74]. The *oprD* sequence data have proven to be an extremely interesting genetic marker, for the following reasons: (i) resistance to carbapenems is often achieved by defective *oprD* mutations (DOMs), (ii) the mosaic structure of the *oprD* gene exposes evidence of recombination events between *P. aeruginosa* strains, (iii) the virtually unlimited number of *oprD* alleles provides high discriminatory power, (iv) despite this extremely high sequence variability, members of narrow clonal complexes often show identical *oprD* sequences, thus illustrating the stability of these complexes [13].

Pyoverdines are high-affinity fluorescent peptidic siderophores secreted by *P. aeruginosa* in order to scavenge Fe(III) in the extracellular environment and shuttle it into the cell [75]. Uptake of the pyoverdine-Fe(III) complex is mediated by FpvA, a specific outer membrane receptor protein. Three *P. aeruginosa* siderovars can be distinguished, each producing a different pyoverdine (type I, II and III) and a matching cognate FpvA receptor [76]–[78]. The type II pyoverdine receptors are more diverse and it has been suggested that they are under positive selection [79]–[80]. Two distinct type II pyoverdine receptor gene clusters were observed: IIa and IIb [81]. In 2004, an additional pyoverdine receptor, FpvB, was discovered [82]. It was found to confer, in pyoverdine type II and III producing *P. aeruginosa* strains, the capacity to utilize type I pyoverdine as a source of iron. The majority of *P. aeruginosa* strains were shown to possess the *fpvB* gene.

ExoS and ExoU are effector molecules (exoenzymes) that can be injected directly into the host cell by the type III secretion system. There are indications that ExoS is the major cytotoxin required for colonization and dissemination during infection, while secretion of ExoU has been associated with increased virulence [83], [84].

The pilin glycosyltransferase TfpO (also called PilO) is an inner membrane protein that captures O antigen subunits and attaches them to a serine residue at the carboxy terminus of the group I pilins [85]. The group I pilin-containing strains can be divided into subgroups: TfpOa (pilin group Ia) strains and TfpOb (pilin group Ib) strains. Analysis of pilin allele distribution among isolates from various sources revealed a striking bias in the prevalence of isolates with group I pilin genes from CF compared with non-CF human sources, suggesting that this particular pilin type may confer a colonization or persistence advantage in the CF host [86].

The above-described traits were combined and analysed using biological data analysis software. The results were visualised using a minimum spanning tree (MST).

Finally, the minimum inhibitory concentrations (MIC) of 21 antimicrobials were determined for the 328 isolates.

## Results and Discussion

### Serotype

**Figure 1:**
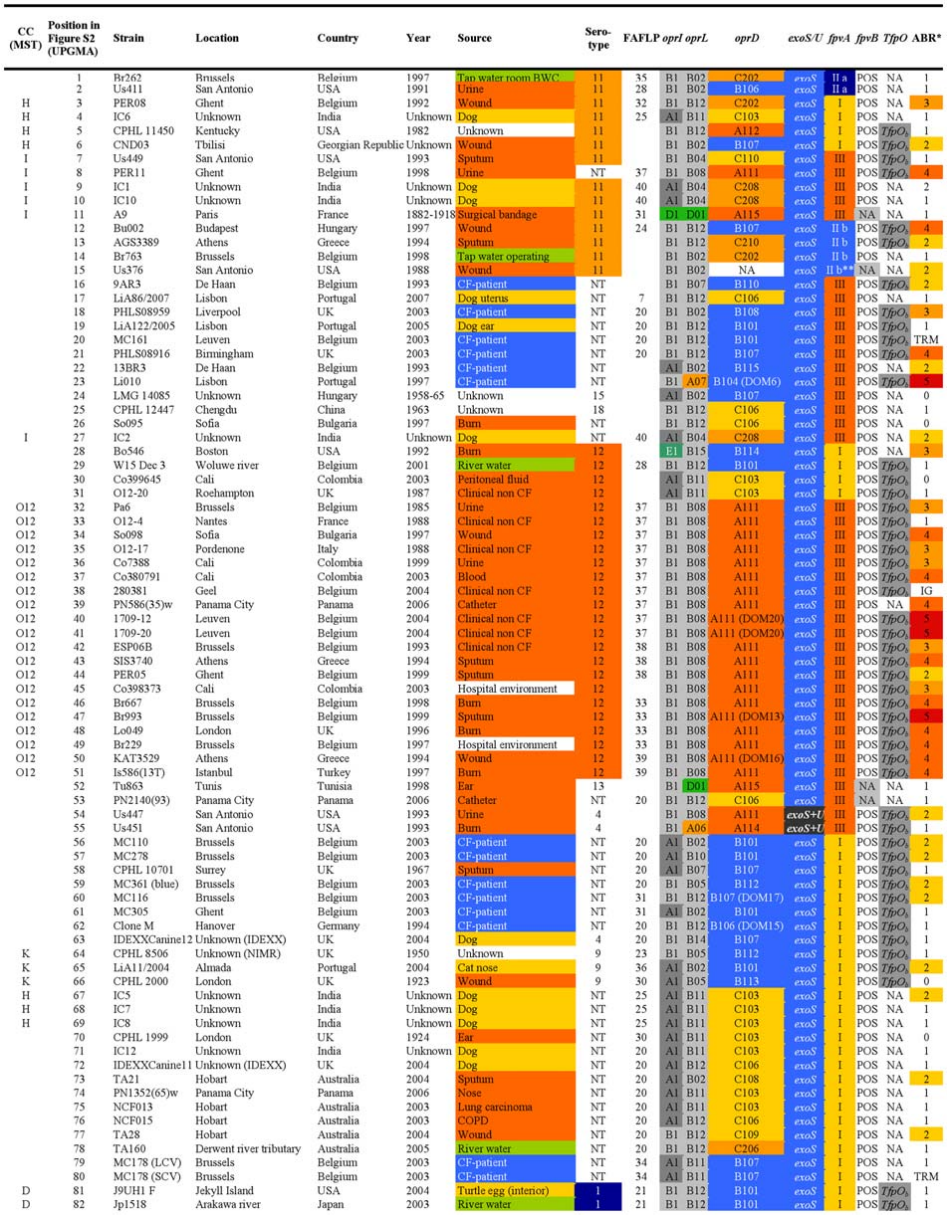
Overview of the characteristics and test results of *P. aeruginosa* strains 1-82/328. ABR: antibiotic resistance; ATCC: American Type Culture Collection; BWC: burn wound centre; CC: clonal complex; CF: cystic fibrosis; COPD: chronic obstructive pulmonary disease; CPHL: Central Public Health Laboratory, London; DOM: defective *oprD* mutation; ESP: Ecole de Santé Publique, Brussels; FAFLP: fluorescent amplified fragment length polymorphism; HPA: Health protection Agency, Colindale; HSV: high sequence variability; IDEXX: laboratory for veterinary, food and water testing; ICU: intensive care unit; IG: insufficient growth; LMG: Laboratorium voor Microbiologie Gent, public bacteria collection; NA: no amplification; NIMR: National Institute for Medical Research, London; NT: not typable; POS: positive; TRM: reaction terminated. * ABR: antibiotic resistance, expressed as the number of antibiotic classes to which resistance was observed. ** Detected using degenerate primers.

**Figure 2:**
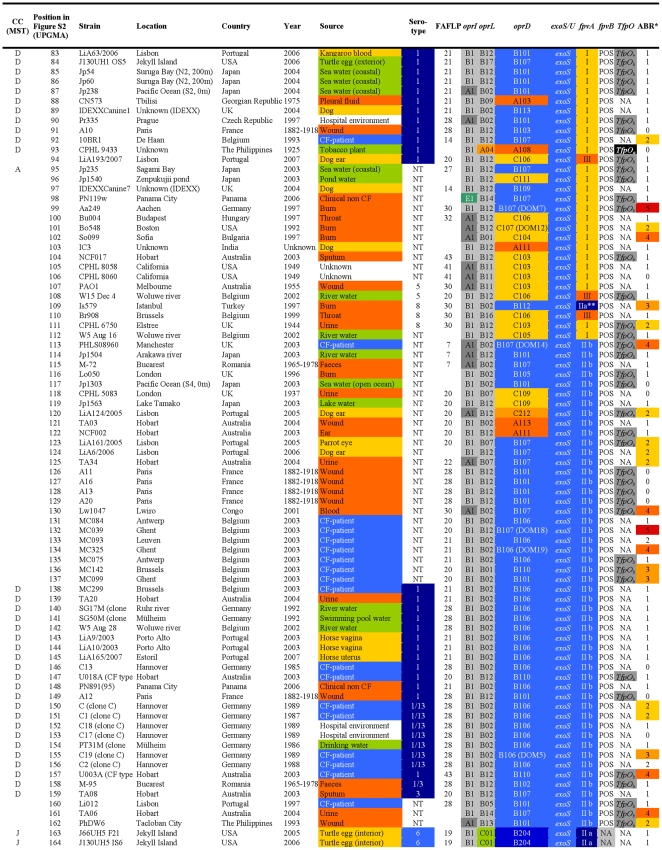
Overview of the characteristics and test results of *P. aeruginosa* strains 83-164/328.

**Figure 3:**
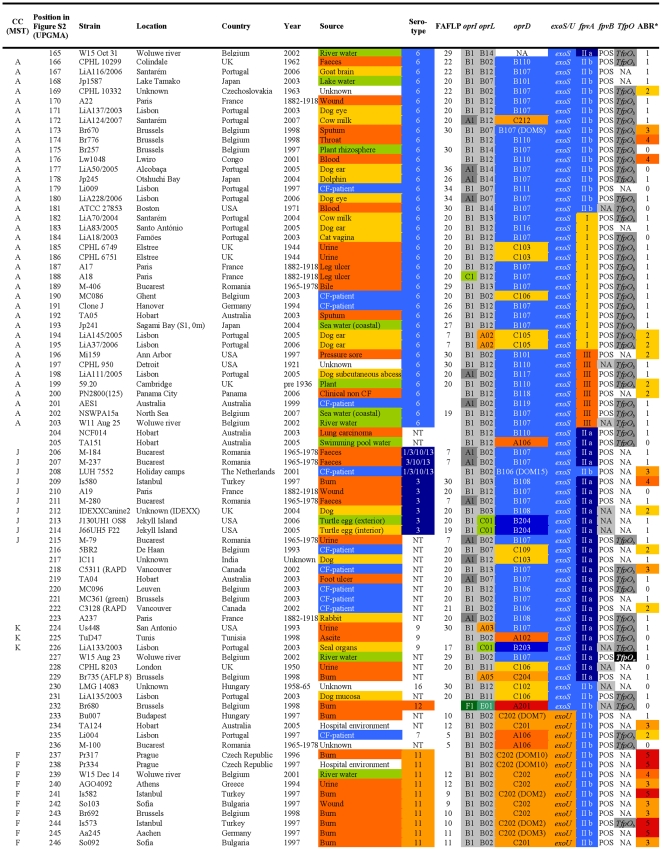
Overview of the characteristics and test results of *P. aeruginosa* strains 165-246/328.

**Figure 4:**
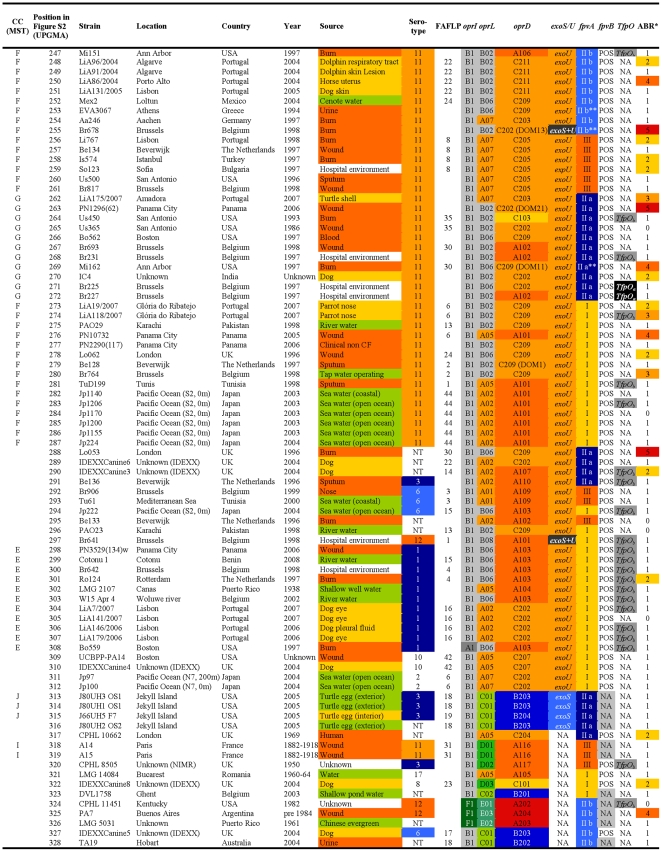
Overview of the characteristics and test results of *P. aeruginosa* strains 247-328/328.

Only 215 (65%) out of the 328 strains could be serotyped (Table 3). This surprisingly low percentage is partially due to the nonagglutinability of 33 out of 43 CF isolates. Additionally, we suspect that the commercially available monoclonal antibody suspensions are not as potent as some of the homemade antisera that were used in past studies. Eleven strains, including 5 CF isolates, were polyagglutinable. Nonagglutinable strains have lost most or all of their lipopolysaccharide (LPS) and polyagglutinable strains have lost part or all of their O-repeating saccharide units, which determine serotype specificity, due to a defective LPS side chain synthesis [87], [88]. Cross-reactions in agglutination are due to core LPS epitopes, which are conserved in all the serotypes. Loss of O serotype reaction was described as one of the distinctive features for P. aeruginosa strains isolated from CF patients with chronic bronchopulmonary infection [89]. Already in 1975 Zierdt and Williams reported that isolates from CF patients were frequently polyagglutinable [90]. The predominant serotypes in our collection were O11 (20.1%), O6 (14.2%), O1 (11.9%) and O12 (7.9%) (Table 3). This is in congruence with the findings of Bert and Lambert-Zechowsky, who determined the O-serotypes of 2952 P. aeruginosa isolates and found serotypes O11, O6 and O1 to be predominant [91]. The incidence of O12, however, was low. The higher prevalence of serotype O12 in our collection is due to an overrepresentation of MDR strains. As could be expected, most MDR clinical isolates exhibited serotypes O11 and O12 (Figure 1, Figure 2, Figure 3, Figure 4, and Table 3). Finally, we would like to stress that the occasional clustering of isolates with different serotypes is not necessarily the result of recombinational events. It was demonstrated that anti-pseudomonal drugs [92] and bacteriophages [93] were able to induce serotype conversion in *P. aeruginosa.*

**Table 3:** Differential prevalence (%) of strain characteristics.

Experiment	Type/group	CF	Clinical non-CF	Animal	Environ-mental	Total*	O11	O12 clone	pre 1980
	Number of strains	43	142	63	55	303	61	20	49
Serotype	O1	5 (11.6)	8 (5.6)	11 (17.5)	12 (21.8)	36 (11.9)	0 (0)	0 (0)	5 (10.2)
	O6	4 (9.3)	15 (10.6)	15 (23.8)	9 (16.4)	43 (14.2)	0 (0)	0 (0)	11 (22.4)
	O11	0 (0)	38 (26.8)	11 (17.5)	12 (21.8)	61 (20.1)	61 (100)	0 (0)	3 (6.1)
	O12	0 (0)	23 (16.2)	0 (0)	1 (1.8)	24 (7.9)	0 (0)	20 (100)	0 (0)
	NT	28 (65.1)	39 (27.5)	18 (28.6)	13 (23.6)	98 (32.3)	0 (0)	0 (0)	16 (32.7)
	PA	5 (11.6)	3 (2.1)	0 (0)	1 (1.8)	9 (3.0)	0 (0)	0 (0)	2 (4.1)
	other	1 (2.3)	16 (11.3)	8 (12.7)	7 (12.7)	32 (10.6)	0 (0)	0 (0)	12 (24.5)
*exoS/U*	*S*	42 (97.7)	98 (69.0)	46 (73.0)	34 (61.8)	220 (72.6)	13 (21.3)	20 (100)	41 (83.7)
	*U*	1 (2.3)	36 (25.4)	15 (23.8)	18 (32.7)	70 (23.1)	45 (73.8)	0 (0)	2 (4.1)
	*S*+*U*	0 (0)	3 (2.1)	0 (0)	0 (0)	3 (1.0)	1 (1.6)	0 (0)	0 (0)
	NA	0 (0)	5 (3.5)	2 (1.6)	3 (5.5)	10 (3.3)	2 (3.3)	0 (0)	6 (12.2)
*oprD*	A	1 (2.3)	41 (28.9)	2 (1.6)	15 (27.3)	59 (19.5)	13 (21.3)	20 (100)	10 (20.4)
	B	40 (93.0)	47 (33.1)	29 (46.0)	25 (45.5)	141 (46.5)	3 (4.9)	0 (0)	27 (55.1)
	C	2 (4.7)	53 (37.3)	32 (50.8)	14 (25.5)	101 (33.3)	44 (72.1)	0 (0)	12 (24.5)
	NA	0 (0)	1 (0.7)	0 (0)	1 (1.8)	2 (0.7)	1 (1.6)	0 (0)	0 (0)
	DOM	7 (16.3)	15 (10.6)	0 (0)	0 (0)	22 (7.3)	9 (14.8)	4 (20.0)	0 (0)
*oprL*	A	1 (2.3)	16 (11.3)	10 (15.9)	12 (21.8)	39 (12.9)	15 (24.6)	0 (0)	4 (8.2)
	B	42 (97.7)	119 (83.8)	46 (73.0)	37 (67.3)	244 (80.5)	43 (70.5)	20 (100)	40 (81.6)
	other	0 (0)	7 (4.9)	7 (11.1)	6 (10.9)	20 (6.6)	3 (4.9)	0 (0)	5 (10.2)
*oprI*	A	8 (18.6)	23 (16.2)	18 (28.6)	2 (3.6)	51 (16.8)	3 (4.9)	0 (0)	13 (26.3)
	B	35 (81.4)	113 (79.6)	45 (71.4)	52 (94.5)	245 (80.9)	57 (93.4)	20 (100)	33 (67.3)
	other	0 (0)	6 (4.2)	0 (0)	1 (1.8)	7 (2.3)	1 (1.6)	0 (0)	3 (6.1)
*fpvA*	I	11 (25.6)	38 (26.8)	26 (41.3)	29 (52.7)	104 (34.3)	18 (29.5)	0 (0)	18 (36.7)
	IIa	5 (11.6)	23 (16.2)	12 (19.0)	8 (14.5)	48 (15.8)	10 (16.4)	0 (0)	8 (16.3)
	IIb	20 (46.5)	43 (30.3)	18 (28.6)	13 (23.6)	94 (31.0)	22 (36.1)	0 (0)	5 (10.2)
	III	7 (16.3)	38 (26.8)	7 (11.1)	5 (9.1)	57 (18.8)	11 (18.0)	20 (100)	8 (16.3)
*fpvB*	POS	43 (100)	131 (92.3)	57 (90.5)	48 (87.3)	279 (92.1)	57 (93.4)	20 (100)	41 (83.7)
	NA	0 (0)	11 (7.7)	6 (9.5)	7 (12.7)	24 (7.9)	4 (6.6)	0 (0)	8 (16.3)
*tfpO*	a	0 (0)	0 (0)	0 (0)	2 (3.6)	2 (0.7)	0 (0)	0 (0)	1 (2.0)
	b	24 (55.8)	70 (49.3)	26 (41.3)	24 (43.6)	144 (47.5)	10 (16.4)	19 (95.0)	27 (55.1)
	NA	19 (44.2)	72 (50.7)	37 (58.7)	29 (52.7)	157 (51.8)	51 (83.6)	1 (5.0)	21 (42.9)

CF, cystic fibrosis; DOM, defective *oprD* mutation; NA, no amplification; NT, not typable; PA, polyagglutinable; POS, positive.

*Strains of unknown origin (n = 12) and strains isolated from the hospital environment (n = 13) were not considered.

### FAFLP

**Figure 5:**
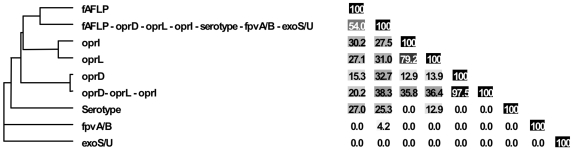
Congruence between experiments, as calculated using the Pearson product-moment correlation coefficient.

The FAFLP patterns of the *P. aeruginosa* strains were normalised and clustered using the Unweighted Pair Group Method with Arithmetic mean (UPGMA). By applying the criteria for differentiation of *P. aeruginosa* by FAFLP [94], which were based on the criteria for pulsed-field gel electrophoresis [95], 44 clusters of related isolates (with ≥80% homology) were identified and numbered (Figure 1, Figure 2, Figure 3, Figure 4, and Figure S1). The close genetic relationship between some isolates, illustrated by an almost identical data set (Figure 1, Figure 2, Figure 3, Figure 4), also resulted in very similar FAFLP patterns (Figure S1). This shows that FAFLP can be used, in clinical settings for example, to recognize epidemic *P. aeruginosa* clones during short time spans. In contrast, the relationship between the different clonal complexes, and sometimes even between distinct clones within a complex, was not always supported by FAFLP (Figure 1, Figure 2, Figure 3, Figure 4). This is illustrated by a congruence of only 54% (lineair correlation) between the similarity matrix of FAFLP and the matrix derived from a combination of all the methods (Figure 5). FAFLP is useful to discriminate between isolates, when investigating local epidemics, but on its own it is not capable to identify clonal complexes and elucidate the population structure of *P. aeruginosa*.

### *ExoS* and *exoU*

Seventy-three percent of all isolates harboured the exoS gene and 23% the *exoU* gene (Table 3). With the exception of three strains, the carriage of *exoU* and *exoS* was mutually exclusive and in 10 isolates neither of the genes could be amplified by PCR (Table 3). Interestingly, 42 of the 43 CF isolates exhibited the *exoS* ^+^/*exoU* ^-^ genotype (Table 3). This could mean that the presence of *exoS*, which is indicative of an invasive phenotype [96], and/or the absence of *exoU*, which has been associated with virulence [83]–[84] and severity of disease [97], is mandatory for successful colonisation of the CF lung. These results are in congruence with earlier reports. Feltman and colleagues observed that 72% of *P. aeruginosa* isolates contained the *exoS* gene and 28% the *exoU* gene [98]. The presence of the *exoS* and *exoU* genes appeared to be mutually exclusive and they also observed that CF isolates harboured more frequently the *exoS* gene and less frequently the *exoU* gene than did isolates from other sites of infection, including the respiratory tract of patients without CF. Wareham and Curtis also observed an association of the *exoS* ^+^/*exoU* ^−^ genotype with chronic infection in CF patients, whilst the *exoS* ^−^/*exoU* ^+^ genotype was associated with strains isolated from blood [99]. The mutual exclusion of *exoS* or *exoU* indicates that selective pressures contributed to the evolution of these genomes in different environmental niches [17]. Because the type III secretion system secretes both ExoS and ExoU, the adaptation to either one of these exoenzymes almost certainly involved interaction with different target eukaryotic organisms. Accordingly, Ferguson *et al*. suggested that in the transition of *P. aeruginosa* from the soil to certain clinical settings, the loss of ExoS expression is favoured [100]. In clinical settings the inactivation of host cell function [101] and the antiphagocytic properties [102] of ExoS should aid in the infectious process, but its limited cytotoxicity, combined with its inefficient targeting of cells of lymphoid origin, may favour the production of more cytotoxic factors, such as ExoU and exotoxin A [103], at certain sites of *P. aeruginosa* infection.

Kulasekara *et al*. suggested that the evolutionary history of the *exoU* locus more than likely involved transposition of the ExoU determinant onto a transmissible plasmid, followed by transfer of this plasmid into different *P. aeruginosa* strains [104]. This is in accordance with our results and would explain the three strains that harbour both *exoS* and *exoU*. The acquisition of novel genetic material, such as the *exoU* genomic island, through HGT, may enhance colonisation and survival in different host environments [17].

### *OprI*, *oprL*, and *oprD*

**Figure 6:**
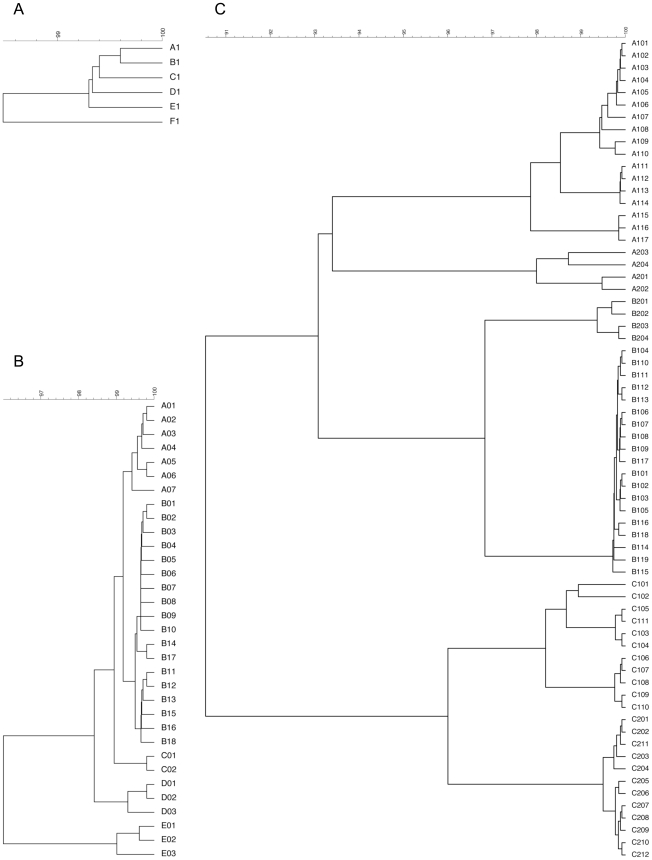
UPGMA dendrograms, with assignment of allele codes, for the *oprI* (a), *oprL* (b), and *oprD* (c) alleles detected in 328 *P. aeruginosa* strains.

The *oprI*, *oprL*, and *oprD* sequences of the 328 studied *P. aeruginosa* strains were aligned and clustered using UPGMA. Allele codes were arbitrarily assigned and consisted of a capital letter for the allele group and a number, according to their position in the alignment (Figure 6). The *oprI* and *oprL* genes showed moderate sequence variability comparable to that of housekeeping genes, as could be expected since both genes code for a structural outer membrane lipoprotein (Table 4). The *oprI*, *oprL* and *oprD* sequences of strains LMG 5031, Br680, CPHL 11451 and PA7 diverged considerably (Figure 6). With the exception of one isolate, all mutations in *oprI* and *oprL* were silent (http://www.pseudomonas.com/related_links.jsp#alleles). All CF isolates but one possessed the group B *oprL* allele (Figure 1, Figure 2, Figure 3, Figure 4, and Table 3). Since non-silent mutations are extremely rare in *oprL*, the conservation of distinct alleles within a clonal complex or clone is likely the result of a genetic linkage or co-selection.

**Table 4:** Analysis of the *oprI*, *L*, and *D* nucleotide sequence data in 328 *P. aeruginosa* strains.

Gene	Size (bp)	No. of alleles	No. of variable sites	% Variable sites	Max. nucleotide distance between alleles (%)
*oprI*	249	6	7	2.8	1.5
*oprL*	504	33	27	5.4	4.0
*oprD*	1323 or 1329	67	274	20.7	9.5

The *oprD* gene showed the expected high sequence variability (Figure 6C and Table 4), typical for a gene that is under strong selection for diversity (http://www.pseudomonas.com/related_links.jsp#alleles). The *oprD* genes of strains US376 and W15 Oct 31 could not be amplified by PCR. The *oprD* gene of these strains is probably not present or exhibits an aberrant nucleotide sequence (at least at the primer annealing sites). With the exception of three isolates (Li004, 5BR2 and MC086), all CF isolates exhibited a group B *oprD* allele (Figure 1, Figure 2, Figure 3, Figure 4, and Table 3). Although genetic linkage or co-selection between group B *oprD* alleles and parameters that have a significant impact on selection in the CF lung are likely, the important intergroup amino acid differences, especially in the external loops of the OprD porin [74], could be indicative of a more decisive role for OprD in the selection for strains in the CF niche. The actual weight of OprD as a selection force in the CF niche cannot be determined from our data and needs further investigation. Twenty-one different defective *oprD* mutations (DOMs), conferring resistance to carbapenem antibiotics, were observed (Table 3 and Table 5). Seven (16.3%) of the 43 CF isolates exhibited a DOM and as a consequence are expected to express no OprD porin. This could mean that if OprD is truly a selective force in the CF niche, it is likely that it will only be of importance in the early stages of colonisation. Additionally, this relatively high percentage of DOMs suggests that carbapenems have an impact on *P. aeruginosa* in the CF lung. Finally, from the congruence chart (Figure 5) we learn that the similarity matrix obtained from the *oprD* data alone is almost identical to that obtained from *oprD*, *oprL* and *oprI* combined, indicating that *oprL* and *oprI* add little or nothing to the discriminatory power of *oprD*.

**Table 5:** Defective *oprD* mutations (DOMs) in 328 *P. aeruginosa* isolates.

DOM	Strain	Mutation
1	Be128	Large deletion starting from NT 874 and covering the termination codon
2	Is573	C→T base substitution at NT 1018 → premature termination
3	Aa245	C→T base substitution at NT 1243 → premature termination
4	Bu007	G→T base substitution at NT 511 → premature termination
5	C19	G→A base substitution at NT 413 → premature termination
6	Li010	G→A base substitution at NT 831 → premature termination
7	Aa249	T→C base substitution at NT 32 → leucine replaced by proline in signal peptide
8	Br670	T→C base substitution at NT 1076 → leucine replaced by proline in external loop 7
9	Br718	Duplication between GCGCGG repeats (NT 573-8 and 617-22) → frameshift → stop codon at NT 730-2
10	Pr317	1-base duplication at monotonic repeat CCCC (NT 346-9) → frameshift → stop codon at NT 352-4
11	Mi162	1-base deletion at monotonic repeat CCCC (NT 346-9) → frameshift → stop codon at NT 379-81
12	Bo548	1-base deletion at monotonic repeat GGGGG (NT 631-5) → frameshift → stop codon at NT 713-5
13	Br993/678	Large deletion covering initiation codon
14	PHLS08960	A→T base substitution at NT 886 → premature termination
15	LUH7552	Duplication of GCC at NT 859-62 → frameshift → stop codon at NT 913-5
16	KAT3529	Deletion of A at NT 1082 → frameshift → stop codon at NT 1294-6
17	MC116	Deletion of AA at NT 820-1 → frameshift → stop codon at NT 1090-2
18	MC039	C→T base substitution at NT 883 → premature termination
19	MC325	Duplication of C at NT 540 → frameshift → stop codon at NT 580-2
20	1709-12	G→A base substitution at NT 195 → premature termination
21	PN1296	Deletion of G at NT 423 frameshift → stop codon at NT 713-5

NT, nucleotide.

### Pyoverdine receptors

No significant correlation could be established between the *fpvA* pyoverdine receptor gene type and habitat (Table 3). De Vos *et al*. reported a prevalence of pyoverdine type II isolates in CF patients and suggested that there might be a correlation between *fpvA* type and the (clinical) origin of the *P. aeruginosa* isolates [105]. We did observe a higher prevalence of pyoverdine type II, and more specifically type IIb (46.5%), in the CF isolates as compared to the total collection (31.0%) (Table 3), but it seems unlikely that the pyoverdine receptor is in itself a selective force in the CF niche.

The relatively unordered distribution of the different pyoverdine receptor types over the different clonal complexes is suggestive for multiple recombinatorial events involving pyoverdine receptors (Figure 1, Figure 2, Figure 3, Figure 4) and a complex evolutionary history. Tümmler and Cornelis reviewed the evolution of the pyoverdine receptor in *P. aeruginosa* and claimed that the pyoverdine region is the most divergent locus of the core genome because it is subject to speciation and coevolution, encodes a trait of altruistic cooperation (the production of siderophores), and encodes a receptor that is both a major fitness allele and a major deleterious allele [79]. Indeed, the mosaic dispersal of *fpvA* types among the different clonal complexes (Figure 1, Figure 2, Figure 3, Figure 4) is possibly the result of the selection pressure caused by bacteriocins, which use the pyoverdine receptors to enter the bacteria. Pyocin S3 was shown to use the type II FpvA receptor, while pyocin S2 was found to kill strains harbouring the type I FpvA receptor [106]–[107].

As expected, the *fpvB* gene was present in the majority (93.4%) of *P. aeruginosa* isolates, including all 43 CF isolates.

### *TfpO*

The *tfpO* gene, indicative for group I pilins, was detected in 48.2% of isolates (Table 3). The *tfpOa* allele was very rare; it was only detected in four isolates (Table 3). The *tfpO* gene was present in 55.8% of CF isolates, which is only slightly higher than the average (48.2%). Thus, in contrast to Kus *et al*. [86] who detected the *tfpO* gene in 69.7% of CF isolates, we did not find a strong association of *tfpO* with CF. The *tfpO* data were found to have only very limited value and discriminatory power and were therefore not included in the combined analysis.

### Population structure

MSTs have long been used in the context of mathematical topology. When a set of distances is given between entries (strains in this case), a minimum spanning tree connects all entries in such a way that the summed distance of all branches of the tree is the shortest possible [108]. In a biological context, this principle adheres to the idea that evolution should be explained in as few events as possible. MST suffers from a serious degree of degeneracy as it generates a large number of solutions, many of which have no biological relevance. Hence, priority rules are applied in order to find or assign the biologically most relevant solution amongst the many solutions. MST analysis was originally developed to link MLVA-derived sequence types (STs) [109], but technically it can be used for any data type, as long as a true distance matrix can be calculated. The MST principle, however, requires that all samples are present in the data set to construct the tree. Internal branches are normally also based upon existing samples. This means that, when an MST is calculated for evolutionary studies, there are two important conditions that have to be met: (1) the study must focus on a short time-frame, assuming that all forms or states are still present, and (2) the sampled data set must be sufficiently complete to enable the method to construct a valid tree, i.e. representing the full biodiversity of forms or states as closely as possible [108]. A major advantage of the MST approach is that the algorithm may result in trees with star-like branches, which allows for a correct classification of population systems that have a strong mutational or recombinational rate, such as *P. aeruginosa*, and where a large number of single locus variants (SLVs) may evolve from one common type [7]. As mentioned above, MSTs can be calculated from a true distance matrix. A distance matrix based upon a data matrix (in the case of fingerprint type data, derived after a global band matching), whether derived from one or multiple data sources, can be used. In theory, every distance coefficient applied on a data matrix produces a distance matrix suitable for analysis with the MST method [108]. Recently MST was used to determine the phylogenetic framework of *Listeria monocytogenes* [110]. In this study MST was used, for the first time, to link the Polyphasic Profiles (PPs) of 328 unrelated *P. aeruginosa* strains in such a way that the sum of the distances (number of differences between two distinct PPs) is minimized.

**Figure 7:**
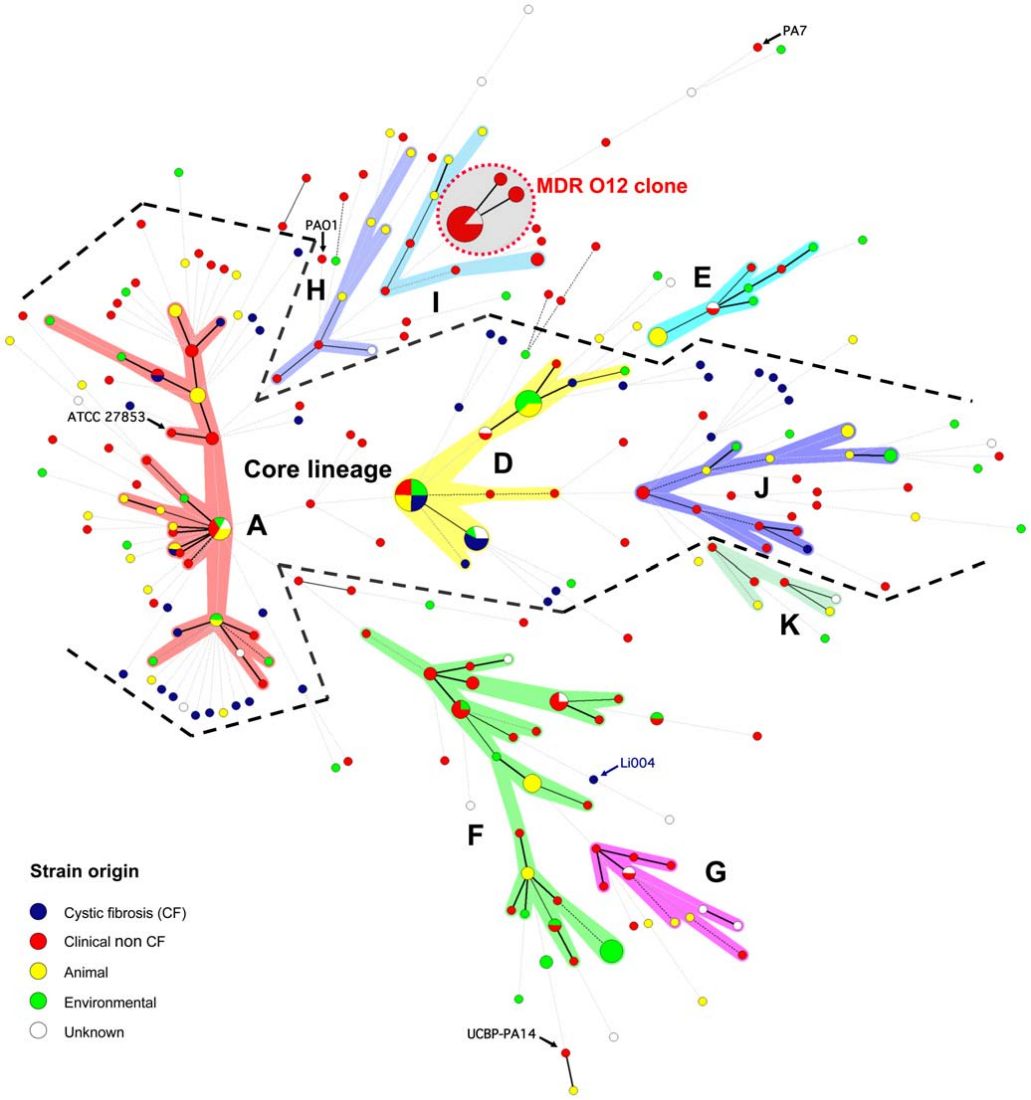
Minimum spanning tree of the similarity matrix of the composite data set consisting of the FAFLP pattern, serotype, *oprI*, *oprL*, and *oprD* gene sequences, pyoverdine receptor profile and prevalence of *exoS/U* for 328 *P. aeruginosa* strains. Each circle corresponds to a polyphasic profile (PP). The circles are scaled with member count. Branch lengths are logarithmic. Coloured zones surround PPs that belong to the same clonal complex. These complexes are also indicated with a capital letter. The lines between PPs indicate inferred phylogenetic relationships and are represented as bold, plain, discontinuous and light discontinuous depending on the number of differences between profile types. Discontinuous links are only indicative. Two bold black indent lines delimit the *P. aeruginosa* “core lineage”; the MDR serotype O12 clone is encircled by a red dotted line.

In our previous *P. aeruginosa* population structure study a UPGMA dendrogram, based on the comparison of the composite data set consisting of 4 markers in 73 strains, revealed 7 distinct clonal complexes, arbitrarily labelled CC A to CC G [13]. In the present MST, based on the composite similarity matrix derived from the combination of 7 markers in 328 strains (Figure 7), we identified 4 additional clonal complexes (CC H to CC K). The former CC C was renamed ‘clone O12’ to avoid confusion with the worldwide CF and aquatic clone C [3] and the former CC B disappeared as its members no longer clustered into a distinct clonal complex. We also observed several distinct isolates with a unique PP, some of which diverged considerably from the rest of the population (e.g. reference strains PA7 and UCBPP-PA14). Strains isolated from inanimate environments, animals and humans, separated by thousands of miles, often clustered into the same clonal complex, confirming that, in general, there is no clear correlation between the clonal complexes and geographical origin or (disease) habitat. As in our previous study, there was again strong evidence that the relation among the isolates was distorted by recombination. We observed a network of relationships between all analysed parameters (Figure 1, Figure 2, Figure 3, Figure 4) and a relatively low congruence between experiments (Figure 5). Evidence of recombination is additionally supported by the mosaic structure of the *oprD* gene (http://www.pseudomonas.com/related_links.jsp#alleles), which is the result of a history of intra and possibly inter species recombinational exchanges of DNA blocks [74]. We also observed several conserved clones, characterized by an almost identical data set (Figure 1, Figure 2, Figure 3, Figure 4) and represented by relatively large numbers of isolates (circles) in the MST (Figure 7). The results of this polyphasic characterization confirm the nonclonal epidemic population structure of *P. aeruginosa*, *i.e.* a superficially clonal structure with frequent recombinations, in which occasionally highly successful epidemic clones arise.

A conventional UPGMA dendrogram based on the composite similarity matrix is shown in Figure S2.

### CF “transmissible” clones

According to this study, a typical CF strain shows the following profile: non- or polyagglutinable (76.5%), *oprD* goup B (93.0%), *oprL* group B (97.7%), *exoS* ^+^ (97.7%) and *fpvB* ^+^ (100%) (Table 3). Although CF isolates exhibited a genetic diversity that was comparable to that observed in other habitats, all of them, with the exception of Li004, clustered in, or were located at the border of what appears to be a large ‘core lineage’ (Figure 7). This ‘core lineage’ seems to be predominant in disease and environmental habitats across the world and is composed of CCs A, D and J (Figure 7). Li004 was isolated from a CF patient in Lisbon (Figure 3), but it remains unclear whether it is an ‘early’ sporadic strain or a ‘late’ persistent strain. All characteristics that were associated with the CF niche (*exoS*, group B *oprL*, group B *oprD*, *fpvA* type II and presence of *fpvB*) in this study were also prevalent in this ‘core lineage’. This supports the argument that not one parameter in itself, but rather a multitude of linked characteristics are responsible for the selection of particular strains in the CF niche.

Although CF strains isolated in different locations across the world were shown to be genotypically non-identical and thus probably not directly related (Figure 1, Figure 2, Figure 3, Figure 4, and Figure 7), they all clustered into the ‘core lineage’. It is thus quite understandable that CF strains isolated in distant places show some level of relatedness, which should however not be confused with clonality. Lanotte *et al*. determined some genetic features of 162 isolates from different ecological origins [111] and found that 3 major genogroups of *P. aeruginosa* isolates were able to colonize CF patients. Unfortunately, due to different choices of typing techniques and strains between studies, we are not able to match these genogroups to our clonal complexes.

Occasional transmission of CF strains in CF clinics and holiday or rehabilitation camps has been reported [37], [112], [113]. Our results indicate that a widespread or global transmission of successful *P. aeruginosa* CF strains is unlikely to have occurred. Our data suggest that strains belonging to the successful ‘core lineage’ are ubiquitous in the natural environment and are therefore more likely to infect CF patients. In 1994, Römling *et al*. observed that clone C, the major clone in the CF population in Germany, was also overrepresented in soil and aquatic habitats, and suggested that the isolation frequency in CF patients simply reflected the distribution of clones in the environment [3].

### MDR serotype O11 and O12 strains

All confirmed MDR O12 strains, showing resistance to one or more representatives of at least 3 antibiotic classes (Figure 1, Figure 2, Figure 3, Figure 4), clustered into a very conserved clone (Figure 7). These strains typically exhibited the following profile: serotype O12 (100%), *oprD* goup A (100%), *oprL* group B (100%), *oprI* group B (100%), *exoS* ^+^ (100%), *fpvB* ^+^ (100%) and *tfpOb* ^+^ (100%) (Figure 1, Figure 2, Figure 3, Figure 4, and Table 3). MDR serotype O11 strains, in contrast, clustered into CCs F, G, H and I (Figure 7) and showed an overall higher genetic divergence (Figure 1, Figure 2, Figure 3, Figure 4, and Table 3). Serotyping of 7089 *P. aeruginosa* strains, isolated in 16 Belgian hospitals in the period from 1977 to 1986, revealed a steady increase of *P. aeruginosa* O12 isolates from 2% in 1982 to 22% in 1986 [114]. The majority of these O12 isolates showed the same distinctive pyocin and phage types, suggesting a high degree of homogeneity within the O12 strains in Belgium. A multicentre European study provided evidence for a common O12 *P. aeruginosa* strain in Europe [41]. In the present study, all MDR O12 strains, isolated between 1985 and 2006 in 9 countries, some of them separated by thousands of miles, were shown to cluster into a very conserved clone exhibiting virtually no divergence after more than 20 years of ‘evolution’ (Figure 1, Figure 2, Figure 3, Figure 4, and Figure 7). This MDR O12 clone consists exclusively of clinical isolates; absolutely no environmental, animal or CF isolates were part of this clone (Figure 7). Furthermore, only recent strains, isolated post 1980, clustered into this clone (Figure 1, Figure 2, Figure 3, Figure 4, and Table 3). These observations could be indicative of a recent, rapid and widespread dissemination. Natural forces are likely to sustain global dispersal of organisms as small and abundant as bacteria [115], but the increased mobility of humans and the simultaneous worldwide increase of high care facilities is likely to have accelerated the dispersal of these MDR epidemic strains. One could state that the MDR O12 clone is a genuine global epidemic clone. Strains can acquire characteristics (e.g. antibiotic resistance determinants), which are advantageous in a specific niche (e.g. an intensive care unit) and this can lead to a rapid clonal expansion. The O12 clone was, to the best of our knowledge, never isolated from the natural environment and it has been suggested that colonised or infected patients might be the primary reservoirs of the prevalent O12 clone [54], [116].

We feel that the emerging MDR O12 clone is an example of a rapid and sustained adaptation of *P. aeruginosa* to a novel environment. Man-made changes to the (hospital) environment, like the introduction of antimicrobials, are affecting the *P. aeruginosa* population structure.

### Conclusion

This present study is to our knowledge the first report of an MST analysis conducted on a polyphasic data set. The population structure of *P. aeruginosa* was determined by means of a combination of seven valuable experiments. Analysis and clustering based on a single experiment broadly conserved the clonal complexes and clones designated in the MST based on the combined experiments (Figure 1, Figure 2, Figure 3, Figure 4). The relationship between these groups of strains, however, varied according to the considered experiment, which is visualized as a mosaic pattern in Figure 1, Figure 2, Figure 3, Figure 4. Therefore, we are convinced that the ultimate or ‘true’ population structure is most faithfully approached combining as many experiments as feasible, which are then again performed on as many unrelated and diverse strains as feasible.

This polyphasic characterization of 328 diverse and unrelated *P. aeruginosa* strains confirmed the nonclonal epidemic population structure of *P. aeruginosa*. Our results also indicate that there are no widespread CF epidemic clones. CF strains are part of a successful and ubiquitous ‘core lineage’ that have infected CF patients from the natural environment and spread through short to medium range transmission between patients in CF clinics and holiday and rehabilitation camps, possibly helped by breaches in basic infection control measures. In contrast, we report the worldwide spread and persistence of MDR clone O12. The excessive use of antibiotics has caused a worldwide ‘unnatural’ selection for multiply resistant or even panresistant *P. aeruginosa* strains.

We hope that the evolutionary framework presented in this study will serve as the basis for more specific studies that will prove helpful in designing public health policies (e.g. segregation of CF patients or not). Additionally, the exchange of standardized data between laboratories and the creation of international reference databases of typed microorganisms should be encouraged. It will enable an efficient monitoring of changes in microbial populations and consequently allow more adequate infection control measures. Knowing a species population structure and evolutionary paths is the cornerstone of strategies aiming to control it. Specialised follow-up papers, based on the evolutionary framework presented here and dealing with some clinically relevant issues, are in preparation.

## Materials and Methods

### *P. aeruginosa* isolates

A total of 328 *P. aeruginosa* clinical and environmental isolates, collected worldwide (69 localities, 30 countries and 5 continents) were examined (Table 1).

Most of them were isolated in the late eighties and nineties, but 49 were isolated before 1980, including 14 *P. aeruginosa* strains isolated at the ‘Institut Pasteur’ in Paris by Carle Gessard [1] and his colleagues in the late 19^th^ century. The studied collection contained 185 human clinical isolates (including 43 CF, 33 burn, 32 wound, 18 urine, 15 sputum, 6 faeces and 5 blood isolates), 63 animal clinical isolates (39 dogs, 6 turtles, 4 horses, 3 parrots, 3 dolphins, 2 cats, 2 cows, 1 kangaroo, 1 goat, 1 rabbit and 1 seal) and 55 environmental isolates (17 sea water, 16 river water, 6 lake water, 5 turtle egg, 4 plant, 3 tap water, 2 swimming pool water and 2 drink water isolates). Geographical origin, isolation site and time and other relevant characteristics of all *P. aeruginosa* isolates can be found in Figure 1, Figure 2, Figure 3, Figure 4.

The *P. aeruginosa* strains were kindly provided by: Dr. A. T. McManus, US Army Institute of Surgical Research, Texas, USA; Dr. L. Ménesi, General Hospital St. Istvan, Budapest, Hungary; Dr. A. Vanderkelen, Dr. S. Jennes, G. Verbeken and D. Schoeters, Queen Astrid Military Hospital, Neder-Over-Heembeek (Brussels), Belgium; Dr. J. A. Clark, Queen Mary's University Hospital, London, England; Dr. A. F. Vloemans, Rode Kruis Ziekenhuis, Beverwijk, The Netherlands; Dr. T. Taddonio, University of Michigan, Michigan, USA; Dr. A. Radke, Klinik für Verbrennungs- und Plastische Wiederherstellungschirurgie, Aachen, Germany; Prof. R. Konigova, Charles University Hospital, Prague, Czech Republic; Dr. R. G. Tompkins, Burns Institute, Shriners Hospital for Children, Boston, USA; Prof. B. Tümmler, Medizinische Hochschule, Hannover, Germany; Dr. M. Caneira, Hospital de Santa Maria, Lisboa, Portugal; Prof. A. Boudabous, Science Faculty, Tunis, Tunisia; Dr. M. Mergeay, Environmental Technology Expertise Centre, Mol, Belgium; Dr. A. E. Lim Jr., St. Scholastica's College of Health Sciences, Tacloban City, Philippines; Prof. O. Hadjiiski, Scientific Institute of Emergency Medicine Pirogov, Sofia, Bulgaria; Prof. K. Taviloglu, University of Istanbul, Istanbul, Turkey; Dr. W. D. H. Hendriks, Zuiderziekenhuis, Rotterdam, The Netherlands; Dr. G. Wauters, University of Louvain, Brussels, Belgium; Dr. O. Vandenberg, Universitair Ziekenhuis St.-Pierre, Brussels, Belgium; Prof. M. Vaneechoutte, University Hospital Ghent, Gent, Belgium; Prof. J. Van Eldere, Catholic University of Leuven, Leuven, Belgium; Dr. U. Römling, Karolinska Institute, Stockholm, Sweden; Dr. L. Roddam and R. Bradbury, University of Tasmania, Hobart, Australia; Dr. T. L. Pitt, Health Protection Agency, London, UK; Dr. A. Leitão, Faculty of Veterinary Medicine, Lisboa, Portugal; Dr. R. W. Brimicombe, Haga Ziekenhuis, Den Haag, The Netherlands; Prof. N. J. Legakis and Dr. P. T. Tassios, National and Kapodastrian University of Athens, Athens, Greece; Prof. J.-M. Meyer, University Louis Pasteur, Strasbourg, France and Dr. M. P. Crespo, Universidad Santiago de Cali, Cali, Colombia; Dr. M. Merabishvili and Dr. Nina Chanishvili, Eliava Institute, Tbilisi, Georgia; Dr. L. Griffiths, Dr. K. Craven and J. Awong-Taylor, Armstrong Atlantic State University, Savannah, US; Prof. M. de Chial, University of Panama, Panama City, Panama; Dr. N. H. Khan, Dr. N. Kimata and Prof. K. Kogure, University of Tokyo, Tokyo, Japan; Dr. D. Armstrong, Monash Medical Center, Melbourne, Australia; A. Catrijsse, Vlaams Instituut voor de Zee, Oostende, Belgium.

Strains LMG 2107, 5031, 10643, and 14083-5 were purchased from the BCCM™/LMG bacteria collection. The 20 ‘CPHL (Central Public health Laboratory) strains’ were purchased from the National Collection of Type Cultures in London (UK). Strain PAO-1 was kindly provided by Dr. C. K. Stover (PathoGenesis Corporation, Seattle, USA). Strain ATCC 27853 was purchased from Gibson Laboratories (USA).

All isolates were grown overnight in Luria-Bertani broth medium (Gibco-BRL-Life Technologies, Belgium) at 37°C on a rotary shaker (150 rpm). The overnight cultures were mixed with equal amounts of sterile 50% (vol/vol) glycerol (Sigma Aldrich, Belgium) in PBS buffer (Sigma Aldrich, Belgium) and stored in duplicate at −80°C.

### FAFLP

FAFLP utilized an ABI 377 automated fluorescence sequencer (Applied Biosystems, Belgium), and the AFLP™ Microbial Fingerprinting Kit (Applied Biosystems) as detailed in the manufacturer's protocols. The enzymes used were T4 DNA ligase, *Eco*RI, and *Tru*9I (all purchased from Roche Diagnostics, Belgium). The primer pair used was *Eco*RI-0[FAM]/*Mse*I-C. GeneScan-500[ROX] internal standard (Applied Biosystems) was co-electrophoresed with each sample in order to allow an accurate calculation of fragment lengths and correction for variation rates and gel distortions. Normalization and fragment sizing were carried out using GeneScan software (Applied Biosystems, Belgium). Band patterns were imported into the BioNumerics v5.1 software (Applied Maths, Belgium) and normalised; parameters used: background subtraction (10% disc diameter), filtering (arithmetic average), band search (minimum profiling 0.5% relative to the maximum value). Cluster analysis was performed by pairwise calculation of the Pearson correlation; the similarity matrix was clustered using the UPGMA algorithm with optimisation: 0%, position tolerance: 1%; uncertain bands were ignored.

### Serotyping

Strains were grown overnight on Luria-Bertani agar medium (Gibco-BRL-Life Technologies) at 37°C. Isolates were serotyped by slide agglutination according to the International Antigenic Typing Scheme (IATS) for *P. aeruginosa* [117], using a panel of 16 type O monovalent antisera (Bio-Rad, Belgium). Some strains had already been serotyped by the strain providers (e.g. isolates LMG 14084 and CPHL 12447, which were shown to exhibit the provisionary O17 and O18 serotypes).

### PCR and sequencing

Strains were grown overnight in Luria-Bertani broth medium (Gibco-BRL-Life Technologies) at 37°C on a rotary shaker (150 rpm). DNA was extracted from the overnight cultures using the High Pure™ PCR Template Preparation Kit (Roche Diagnostics) according to the manufacturer's guidelines. The complete *oprI*, *oprL*, and *oprD* genes and a fragment of the *exoS*, *exoU*, *fpvA*, *fpvB* and *tfpO* genes were amplified by PCR, using the primers described in Table 6. PCR was performed in 200-µl microcentrifuge tubes. The PCR mixture (50 µl final volume) contained the following: 25.5 µl sterile distilled water, 5 µl 10×PCR buffer (500 mmol/l KCl and 100 mmol/l Tris-HCl: pH 8.3), 4 µl of a deoxynucleotide mixture (dGTP, dTTP, dATP, and dCTP; 2 mmol/l each), 5 µl MgCl_2_ (2.5 mmol/l), 5 µl of a primer mixture (10 µmol/l each), 5 µl template DNA, and 0.5 µl AmpliTaq DNA polymerase (5 U/µl). All PCR-reagents and primers were ordered from PE-Applied Biosystems. The amplification was performed in a GeneAmp® 9700 thermocycler (Applied Biosystems). The amplification program was set at 50 cycles of denaturation at 94°C for 30 s, annealing at a temperature in accordance to the primers (Table 6), for 30 s, and elongation at 72°C for 1 min. In a few strains the amplification of the *fpvA* gene required degenerate primers. The reaction mixture was put on an agarose gel of 1.5 % (wt/vol) for electrophoresis and visualization of the PCR-product after staining with ethidium bromide on a transilluminator. Prior to the sequencing of the *oprD*, *oprL* and *oprI* genes, the respective PCR-products were purified, using centricon® 100 micro-concentrators (Millipore, Brussels, Belgium) according to the manufacturer's instructions. Five µl of the purified PCR fragment was used as a template in the sequencing reaction. PCR primers were used for sequencing. Sequencing of the coding and anti-coding strand of the *oprD* PCR products necessitated two additional internal primers (Table 6). DNA sequencing utilized an ABI 377 automated fluorescence sequencer (Applied Biosystems), and the ABI Prism® BigDye™ Terminator cycle sequencing kit (Applied Biosystems) as detailed in the manufacturer's protocols. The *oprD* gene of isolate Be128 was sequenced directly from genomic DNA. Some genes were sequenced in the VIB Genetic Service Facility (Belgium) using a capillary Applied Biosystems 3730 DNA Analyzer. PCR and sequencing were performed in duplicate in order to be able to detect eventual PCR mistakes.

**Table 6:** Primers for PCR and sequencing.

Target	Primer	Sequence (5′ to 3′)	Tm (°C)	Size (bp)	Reference
*oprI*	PS1	ATGAACAACGTTCTGAAATTCTCTGCT	57	248	[70]
	PS2	CTTGCGGCTGGCTTTTTCCAG			
*oprL*	PAL1	ATGGAAATGCTGAAATTCGGC	57	504	[70]
	PAL2	CTTCTTCAGCTCGACGCGACG			
*oprD*	DF1	ATGAAAGTGATGAAGTGGAGC	49	1323-9	[13]
	DR1	CAGGATCGACAGCGGATAGT			
*oprD* (for sequencing)	DF2	AACCTCAGCGCCTCCCT	49	NA	[13]
	DR2	AGGGAGGCGCTGAGGTT			
*fpvA* I	*fpvA*If	CGAACCCGACGAAGGCCAGA	52	324	[81]
	*fpvA*Ir	GTAGCTGGTGTAGAGGCTCAA			
*fpvA* IIa	*fpvA*IIaf	TACCTCGACGGCCTGCACAT	52	908	[81]
	*fpvA*IIar	GAAGGTGAATGGCTTGCCGT			
*fpvA* IIb	*fpvA*IIbf	GAACAGGGCACCTACCTGTA	52	863	[81]
	*fpvA*IIbr	GATGCCGTTGCTGAACTCGTA			
*fpvA* III	*fpvA*IIIf	ACTGGGACAAGATCCAAGAGA	52	505	[81]
	*fpvA*IIIr	CTGGTAGGACGAAATGCGA			
*fpvB*	*fpvB*f	GCATGAAGCTCGACCAGGA	52	562	[81]
	*fpvB*r	TTGCCCTCGTTGGCCTTG			
*exoS*	exoSF	TCAGGTACCCGGCATTCACTACGCGG	55	572	[98]
	exoSR	TCACTGCAGGTTCGTGACGTCTTTCTTTTA			
*exoU*	exoUF	AGCGTTAGTGACGTGCG	55	1572	[98]
	exoUR	GCGCATGGCATCGAGTAACTG			
*tfpO_a_*	tfpOup	CGTACTATTCTATTATTGCTGA	55	849	[86]
	tfpOdown	CAAAGGATGGGCTACGAA			
*tfpO_b_*	tfpO2up	CTGATGCTGTTTTCCTTC	55	551	[86]
	tfpO2down	GCATCTCGCCACAACACG			

Tm: annealing temperature.

Using the BioNumerics v5.1 software, sequences were grouped via a pairwise clustering (pairwise alignment parameters: open gap penalty: 100%, unit gap penalty: 0%, min. match sequence: 2, max n° of gaps: 9, fast algorithm). The obtained UPGMA tree was used to seed a multiple alignment (multiple alignment parameters: open gap penalty: 100%, unit gap penalty: 0%, min. match sequence: 2, max n° of gaps: 98). Finally, multiple aligned sequences were clustered using the same parameters as used in the initial pairwise clustering, resulting in the final UPGMA tree.

### Conbined data analysis

A data set consisting of the serotype, FAFLP pattern, *oprI, oprL,* and *oprD* gene sequences, pyoverdine receptor profile (*fpvA* and *fpvB*) and prevalence of the genes *exoS* and *exoU* of 328 *P. aeruginosa* isolates was analyzed using the biological data analysis software BioNumerics v5.1. The settings used for the comparison of the FAFLP fingerprints and the gene sequences are described in the respective paragraphs. Serotype, pyoverdine receptor profile and presence of *exoS/U* were compared using the Pearson correlation. These individual comparisons resulted in individual similarity matrices, which were averaged into the similarity matrix of the composite data set. No correction for internal weights was applied. Each isolate was thus assigned a ‘polyphasic profile’ (PP) contributing to the composite similarity matrix. Grouping of the averaged composite similarity matrix was achieved by MST analysis using BioNumerics v5.1 software. The MST coefficient was taken from the composite similarity matrix. The Degeneracy of the MST was reduced through the use of a priority rule by which types that had a maximum number of entries were linked first, confirming a biological meaning that these clones are most likely older. For visual purposes, isolates were further grouped into clonal complexes. For the creation of the clonal complexes, the similarity bin size (1 change) was set to 2.5%; the maximal neighbour distance between two complexes was 5 changes (12.5%) and the minimum size of a complex was 5 types. Originally a clonal complex was defined as a cluster of STs in a burstdiagram in which all STs are linked as SLVs to at least one other ST. In our case a clonal complex is a cluster of PPs, after MST analysis, in which all PPs with less than 5 changes ( =  less than 12.5% distance in the similarity matrix) are linked. Congruence between experiments was calculated using the Pearson product-moment correlation coefficient between the respective similarity matrices.

### Antimicrobial susceptibility tests

Strains were grown 18–24 h at 37°C on Columbia agar containing 5% horse blood (bioMérieux). Suspensions of these cultures were made in 0.45% saline, adjusted to the turbidity of a 0.6 McFarland standard, and used to load the test cards for VITEK 2 (bioMérieux), which was used in accordance with the manufacturer's directions. The following antibiotics were tested using the AST-N020 antimicrobial susceptibility cards: AMP, ampicilin; AMC, amoxicillin + clavulanic acid; PIP, piperacillin; TZP, piperacillin + tazobactam; CEF, cephalothin; CXM, cefuroxime; CTX, cefotaxime; CAZ, ceftazidime; CPD, cefpodoxime; FOX, cefoxitin; FEP, cefepime; MEM, meropenem; GEN, gentamicin; TOB, tobramycin; AMK, amikacin; NOR, norfloxacin; OFX, ofloxacin; CIP, ciprofloxacin; NIT, nitrofurantoin; SXT, trimethoprim + sulfamethoxazole. Antibiotic resistance phenotypes, represented by the minimum inhibitory concentrations (MICs) for the above-mentioned antibiotics, were determined using VITEK 2 Advanced Expert System (AES) [118]. *P. aeruginosa* ATCC 27853 was used as control strain. For some isolates the MIC was determined by the broth microdilution method [119].

### Nucleotide sequences

The nucleotide sequences generated in this study have been deposited in the *Pseudomonas aeruginosa* Genome database (http://www.pseudomonas.com/related_links.jsp#alleles).

### Strain collection

All studied *P. aeruginosa* strains were deposited in the Belgian Coordinated Collections of Microorganisms (BCCM) of the Laboratorium voor Microbiologie (LMG) of the Ghent University. Strains were assigned a BCCM/LMG number (LMG 24881 - 25202). Strains that were obtained from a culture collection (BCCM/LMG or ATCC) maintained their original reference number.

Strains can be obtained from the LMG bacteria collection for research use only and with the consent of the strain donors.

## Supporting Information

Figure S1 UPGMA dendrogram of the FAFLP patterns of the 328 studied *P. aeruginosa* strains.
(0.42 MB PDF)

Figure S2 UPGMA dendrogram of the similarity matrix of the composite data set consisting of the serotype, FAFLP pattern, *oprI*, *L*, and *D* gene sequences, pyoverdine receptor profile and prevalence of *exoS/U* genes for the 328 studied *P. aeruginosa* strains.
(0.02 MB PDF)
