# Supplementary figures and images for: Pseudomonas aeruginosa Population Structure Revisited
Source: PLoS One. 2009 Nov 13;4(11):e7740. doi: 10.1371/journal.pone.0007740 (PMC2777410; doi:10.1371/journal.pone.0007740)

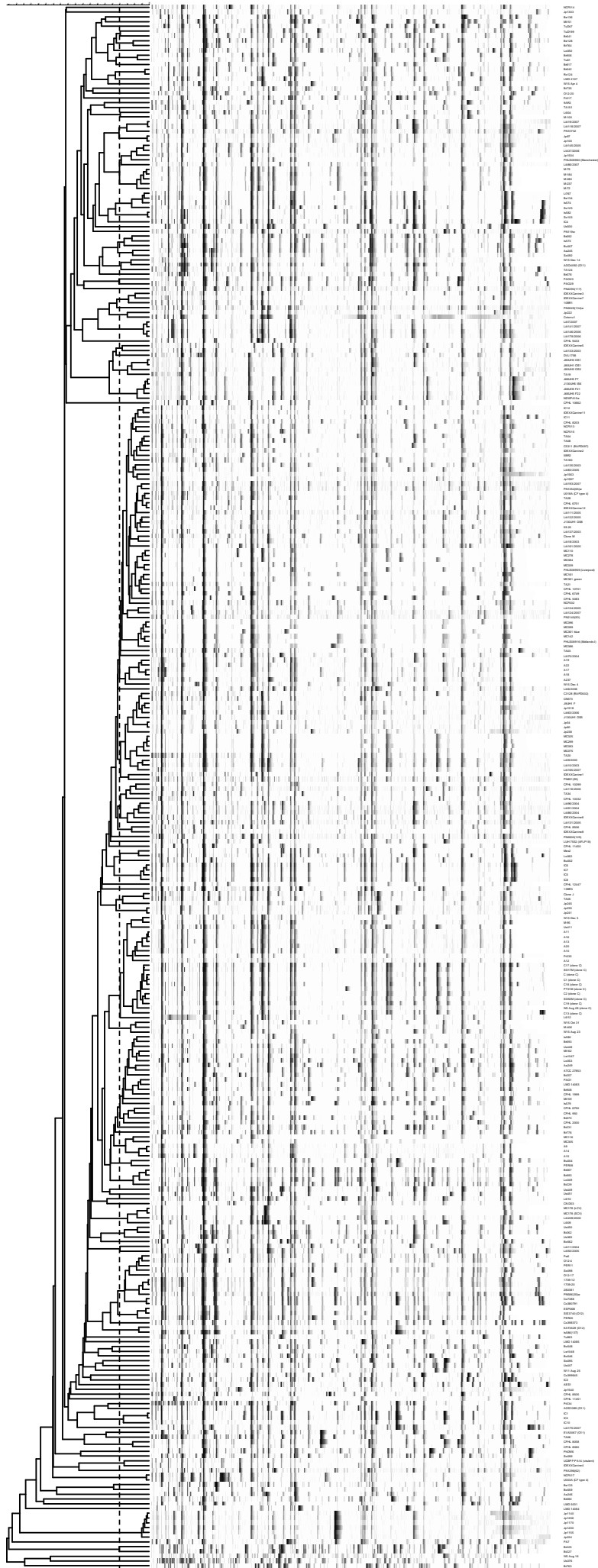

Supplement: Figure S1 — UPGMA dendrogram of the FAFLP patterns of the 328 studied P. aeruginosa strains. (0.42 MB PDF) [file pone.0007740.s001.pdf]
