# Supplementary material for: Pseudomonas aeruginosa Population Structure Revisited
Source: PLoS One. 2009 Nov 13;4(11):e7740. doi: 10.1371/journal.pone.0007740 (PMC2777410; doi:10.1371/journal.pone.0007740)

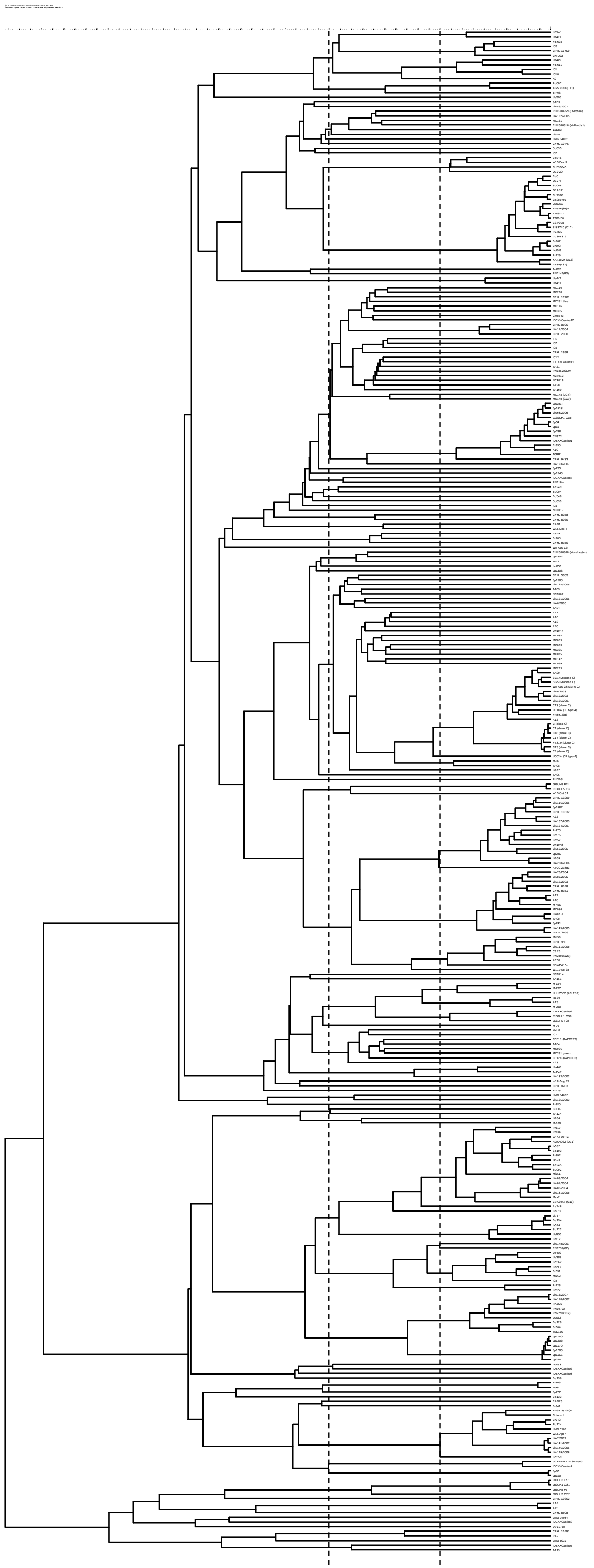

Supplement: Figure S2 — UPGMA dendrogram of the similarity matrix of the composite data set consisting of the serotype, FAFLP pattern, oprI, L, and D gene sequences, pyoverdine receptor profile and prevalence of exoS/U genes for the 328 studied P. aeruginosa strains. (0.02 MB PDF) [file pone.0007740.s002.pdf]
